# An assessment of policymakers’ engagement initiatives to promote evidence informed health policy making in Nigeria

**DOI:** 10.11604/pamj.2017.27.57.9844

**Published:** 2017-05-24

**Authors:** Chigozie Jesse Uneke, Issiaka Sombie, Namoudou Keita, Virgil Lokossou, Ermel Johnson, Pierre Ongolo-Zogo

**Affiliations:** 1 Knowledge Translation Platform, African Institute for Health Policy and Health Systems, Ebonyi State University, PMB 053 Abakaliki Nigeria; 2 Organisation Ouest Africaine de la Santé, 175, avenue Ouezzin Coulibaly, 01 BP 153 Bobo-Dioulasso 01, Burkina Faso; 3 Hopital Central Yaounde, CDBPH Lawrence VERGNE Building 2nd Floor, Avenue Henry Dunant Messa Yaoundé

**Keywords:** Policymakers, evidence informed, health policy, Nigeria

## Abstract

In most developing countries including Nigeria, one of the most challenging issues associated with evidence-to-policy link is the capacity constraints of policymakers to access, synthesize, adapt and utilize available research evidence. The purpose of this review is to assess the efforts and various initiatives that have been undertaken to deliberately engage policymakers and other stakeholders in the health sector in Nigeria for the promotion of evidence informed policymaking. A MEDLINE Entrez Pubmed search was performed and studies that investigated policy making process, evidence to policy link, research to policy mechanism, and researchers/policymakers interaction in Nigeria in relation to health policy were sought. Of the 132 publications found, 14(10.6%) fulfilled the study inclusion criteria and were selected and included in the review. Of the fourteen scientific publications identified, 11 of the studies targeted both researchers and policymakers and the principal tool of intervention was training workshops which focused on various aspects of evidence informed policymaking. All the studies indicated positive outcomes and impacts in relation to quantifiable improvement in policymakers' knowledge and competence in evidence to policy process. Capacity strengthening engagement mechanism is needed for both researchers to generate better evidence and for policymakers and health-care professionals to better use available evidence.

## Introduction

The process of utilizing evidence from research to make health policy which is known as evidence-informed policy-making is characterized by the systematic and transparent access to, and appraisal of, evidence as an input into policy-making [1, 2]. In evidence-informed policy-making, there is a shift away from opinion-based policies and practices to a more rigorous, rational approach that gathers, critically appraises, and uses high-quality research evidence to inform health policy-making, professional practice, and systems operations [3]. Numerous reports from previous investigations have provided convincing information which proves that evidence from research can enhance health policy process and development by identifying new issues for the policy agenda, informing decisions about policy content and direction and evaluating the impact of policy [4–7]. Currently there is a global recognition that strong and effective health systems that are evidence-based in their operations are vital to achieve continued improvement in health outcomes in an efficient and equitable manner [8, 9]. According to World Health Organization (WHO), better use of research evidence in development policy making can save lives through more effective policies that respond to scientific and technological advances, use resources more efficiently and better meet citizens' needs [10]. In most developing countries including Nigeria, one of the most challenging issues associated with evidence-to-policy link is the capacity constraints of policymakers to access, synthesize, adapt and utilize available research evidence [11, 12]. A major factor responsible for this is the lack of engagement/involvement of policymakers in the evidence generation process. In a previous report, Dawad and Veenstra [13] noted that without adequate capacity, in knowledge translation/management and health policy research, policymakers will not have the capacity to access and synthesize sound information on which to base decisions and the potential for shared learning will be lost. According to Green and Bennett [14], knowledge and skill constraints associated with accessing evidence from various sources and competency in making use of the evidence appropriately are among the most important capacity needs of policymakers. It is important to note that capacity strengthening to enhance evidence to policy process will undoubtedly require sustainable platforms and mechanisms that will bring both policymakers and researchers together for interaction [13, 15].

According to Lavis and colleagues [16], there is growing interest in identifying interactive knowledge-sharing mechanisms that allow research evidence to be brought together with the views, experiences and tacit knowledge of those who will be involved in, or affected by, future decisions about high-priority issues. This interest has been fuelled by the recognition of the need for locally contextualized ´decision support´ for policymakers and other stakeholders [17, 18]. This locally contextualized decision support mechanism is one of the main capacity gaps that require to be bridged especially in low and middle income countries (LMICs) where health systems are weak and policies are hardly evidence informed. Deans and Ademokun [19] had noted in their report that those who seek to build capacity for evidence-informed policy need to understand the actual capacity gaps of policy-makers. Furthermore, Green and Bennett [14] had argued that to achieve evidence informed policy making in any area of the health improvement, policy-makers and their advisers, need a set of skills to enable them to use research in their decision-making. They also noted that in particular, policy-makers need to be able to: identify situations where research can help; articulate research questions for topics of policy-relevant research; and access and assess research findings and incorporate them in decision making [14]. Thus, there is a need to strengthen institutions and mechanisms that can more systematically promote interactions between researchers, policy-makers and other stakeholders who can influence the uptake of research findings [20]. The purpose of this review is to assess the efforts and various initiatives that have been undertaken to deliberately engage policymakers and other stakeholders in the health sector in Nigeria for the promotion of evidence informed policymaking. This is with the view to providing scientific information required to build sustainable interactive mechanisms and platforms between those who generate research evidence (researchers) and those in the position to use the evidence generated for decision making (policymakers and implementers).

## Methods

A MEDLINE Entrez Pubmed search was performed in August 2015 and studies published in English that investigated policy making process, evidence to policy link, research to policy mechanism, and researchers/policymakers interaction in Nigeria in relation to health policy were sought. The keywords used for the search included: Nigeria, evidence, health policy; these yielded 132 entries. These 132 publications were subjected to the study inclusion criteria which included the following: (i) must have been conducted in Nigeria; (ii) must be a primary scientific investigation and not a review article; (iii) must target policymakers and researchers or only policymakers; (iv) must address health issue of policy relevance to Nigeria; (v) must produce evidence that is policy relevant; (vi) may of may not have an intervention component. Of the 132 publications found, a total of 14(10.6%) fulfilled these study inclusion criteria and were selected and included in this review [21–34] (Table 1, Table 2, Table 3, Table 4). The selected publications were categorized according to the following information: Author/year of publication; Study methods/key activities; Primary study subjects/targets; health issue investigated; evidence-based intervention; evidence produced from study and policy relevant conclusion (Table 1, Table 2, Table 3, Table 4). The references of the selected publications were reviewed for the identification of studies that may provide additional vital information for this paper.

**Table 1:** Profile and characteristics of scientific publications in 2015 associated with policymakers and evidence to policymaking process in Nigeria

S/No	Author/year of publication/reference	Study methods/key activities	Primary study subjects/targets	Health issue investigated	Evidence-based intervention	Evidence produced from study & Policy relevant conclusion
1	Etiaba et al. 2015[21]	Document reviews and in-depth interviews	Researchers/policymakers	Oral health policy	No intervention implemented in the study	Availability of evidence & socio-political contexts influence evidence
2	Hawkes et al. 2015[22]	Capacity building to increase access to research/data	Researchers/policymakers	Strengthening the capacity of policy makers to promote the use of evidence/data in policy making.	Training workshops	Sustainability of evidence-informed policy making requires strengthening institutional capacity, as well as understanding and addressing the political environment, and incentives facing policy makers that support the use of evidence in policy cycles
3	Uneke et al. 2015[23]	Capacity building & knowledge translation activities	Researchers/policymakers	Implementation of a health policy advisory committee HPAC as a knowledge translation platform	Training workshops, multi-stakeholders policy dialogue	A HPAC can function as a KT platform and can introduce a new dimension towards facilitating evidence-to-policy link into the operation of the MoH, and can serve as an excellent platform to bridge the gap between research and policy

**Table 2:** Profile and characteristics of scientific publications in 2013 associated with policymakers and evidence to policymaking process in Nigeria

S/No	Author/year of publication/reference	Study methods/key activities	Primary study subjects/targets	Health issue investigated	Evidence-based intervention	Evidence produced from study & Policy relevant conclusion
1	Uneke et al. 2013[24]	Evidence-to-policy research priority setting meeting	Researchers/policymakers	Research priority setting	Training workshops	Research priority setting exercise involving policymakers is an example of demand driven strategy in the health policymaking process capable of reversing inequities and strengthening the health systems
2	Moat et al. 2013[25]	Multi-stakeholder deliberative dialogues	Researchers/policymakers	Evaluation of evidence briefs and deliberative dialogues used in the support of evidence-informed policy-making.	Training workshops, multi-stakeholders policy dialogue	Although some aspects of their design may need to be improved, evidence briefs and deliberative dialogues appear to be highly regarded and to lead to intentions to act.
3	Uneke et al. 2013[26]	Cross-sectional intervention study	Researchers/policymakers	Health sector reforms for health systems strengthening	Training workshops	Efforts need to be intensified to enhance competencies of policy makers to adopt an evidence-informed process in health reform programs. Inputs from the public must be given adequate consideration in addressing the challenges of health systems.

**Table 3:** Profile and characteristics of scientific publications in 2012 associated with policymakers and evidence to policymaking process in Nigeria

S/No	Author/year of publication/reference	Study methods/key activities	Primary study subjects/targets	Health issue investigated	Evidence-based intervention	Evidence produced from study & Policy relevant conclusion
1	Uneke et al. 2012[27]	Cross-sectional intervention study	Researchers/policymakers	Leadership and governance competencies to strengthen health systems	Training workshops	More systematic and standardized processes are required to improve competencies of leadership and governance for better human resources development
2	Uneke et al. 2012[28]	Descriptive study	Researchers/policymakers	Role of a health policy advisory committee in bridging the divide between research and policy	Training workshops	Although the primary goal of a HPAC is to promote evidence informed policymaking, the scope of the HPAC’s operation might be expanded to operating as a Knowledge Translation Platform (KTP).
3	Uneke et al. 2012[29]	Cross-sectional intervention study	Researchers/policymakers	Bridging the gap between researchers and policymakers.	Training workshops	Involving policymakers and researchers in planning and execution of health research and health programmes and promoting dialogue between researchers and policymakers can bridge the gap between both parties

**Table 4:** Profile and characteristics of scientific publications in 2008-2010 associated with policymakers and evidence to policymaking process in Nigeria

S/No	Author/year of publication/reference	Study methods/key activities	Primary study subjects/targets	Health issue investigated	Evidence-based intervention	Evidence produced from study & Policy relevant conclusion
1	Uneke et al. 2010[30]	Cross-sectional intervention study	Researchers/policymakers	The challenges and the potential intervention strategies to health policy & systems research in policy making.	Training workshops	Partnership between researchers and policy makers, improvement of staff incentives and facilities for research activities, and sustainable institutional capacity development.
2	Okonofua et al. 2011[31]	Cross-sectional intervention study	Policymakers	An advocacy program aimed at implementing a policy of free maternal and child health (MCH) services	Advocacy and public health education	Advocacy and public health education is effective in increasing the commitment of policymakers to provide resources for implementing evidence-based maternal and child health services in Nigeria.
3	Okonofua et al. 2009[32]	In-depth interviews	Policymakers	Perceptions of policymakers toward unsafe abortion and maternal mortality	No intervention implemented in the study	Strategies to reduce maternal mortality include facilitating access to contraceptives, providing sexuality education, improving the health care system, empowering women and providing free pregnancy care.
4	Garuba et al 2009[33]	Semi-structured interviews using questionnaire	Policymakers & stakeholders in the pharmaceutical system	Perceived level of transparency and potential vulnerability to corruption in pharmaceutical sector	No intervention implemented in the study	Deficiencies include the absence of conflict of interest guidelines, the inconsistency in documentation of procedures, lack of public availability of such documentation, and inadequacies in monitoring and evaluation.
5	Syed et al. 2008[34]	Cross-sectional intervention study	Researchers/policymakers	Exploration of the research-policy interface	Workshops and electronic communications.	Health systems research proposals in low and middle income countries should include reflection on transferring research findings into policy.

### Current status of knowledge

**Knowledge transfer/exchange process involving Nigeria policymakers is recent:** All the studies which fulfilled the study inclusion criteria were published within the eight years (2008-2015). The outcome of this review clearly suggests that research on evidence-informed policymaking and knowledge transfer/exchange processes involving policymakers is new or still at infancy stage in Nigeria. Out of the 14 scientific publications that fulfilled the study inclusion criteria, 9(64.3%) of them were published between 2012 and 2015 (Table 1,Table 2, Table 3). Also only five research teams [21–23, 31,34] have undertaken scientific research that involved the deliberate engagement of the policymakers for capacity enhancement for evidence-informed policymaking and knowledge transfer/exchange. Sutcliffe and Court [35], noted in their report that using evidence to inform policy is not really a new idea, and that what is new and interesting is the increasing emphasis that has been placed on the concept in recent times. This explains why the concept is quickly gaining so much recognition globally and so it is not unexpected for policymakers and researchers in Nigeria to be aware of the concept as was observed in our previous studies conducted in Nigeria [29, 30,36–39].

### Training workshops as principal tool of intervention

Of the fourteen scientific publications identified, 11 of the studies targeted both researchers and policymakers [22–31,34], and the principal tool of intervention was training workshops which focused on various aspects of evidence informed policymaking such as: (i). political environment, incentives facing policy makers that support the use of evidence in policy cycles; (ii). function of a Knowledge Translation platform to bridge the gap between research and policy; (iii). Research priority setting exercise for reversing inequities and strengthening the health systems; (iv). design and use of evidence briefs and deliberative dialogues to improve evidence-to-policy link; (v). enhancing competencies of policy makers to adopt an evidence-informed process in health reform programs; (vi). standardized processes required to improve competencies of leadership and governance; (vii). Partnership building between researchers and policy makers, (viii). Improvement of staff incentives and facilities for research activities, (ix). Health systems research proposals (Table 1,Table 2,Table 3,Table 4). It is of interest to note that up to 11 of the 20 studies targeted both researchers and policymakers and brought them together to participate in interventional training workshops designed to enhance capacity for evidence informed policymaking. Also of interest is the fact that the training workshops addressed vital evidence-to-policy link issues such as political environment and the use of evidence in policy cycles; function of knowledge translation platforms to bridge the gap between research and policy; research priority setting exercise for reversing inequities and strengthening the health systems; design and use of evidence briefs and deliberative dialogues to improve evidence-to-policy link etc. The outcomes of these workshops clearly showed remarkable improvements in the skill and knowledge of the participants regarding evidence-to-policy link. Training workshops of this sort have been reported to have many strategic benefits. The report of healthcare information for all (HIFA) [40] and Poulos and colleagues [41] highlighted some of the benefits of training workshop (when used as in-service training) to include presenting new information to groups of people, practicing new skills and allowing health policymakers and other stakeholders to share experiences and insights. According to Choi and colleagues [42], scientists could become “policy sensitive” through training and participation in the policy-making process, while policy-makers could be exposed to science through training and participation in the research process so they can apply a “science lens” to policy-making. This would promote communication among the policy-makers and researchers by creating a common language and which can help the policy-making process more effective [43,44].

**Quantifiable improvement in policymakers' knowledge and competence in evidence to policy process:** Most of the studies reporting policymakers' capacity enhancement process for evidence informed policymaking were mostly recent ranging from 2012-2015 (Table 1, Table 2, Table 3). All these studies indicated positive outcomes and impacts in relation to quantifiable improvement in policymakers' knowledge and competence in evidence to policy process. Six of the selected studies were cross sectional intervention studies [26,27, 29, 30, 31, 34]. In one of the scientific publications it was noted that sustainability of evidence-informed policy making requires strengthening institutional capacity, as well as understanding and addressing the political environment, and incentives facing policy makers that support the use of evidence in policy cycles [22] (Table 1). Although the studies reviewed did not assess the long term impact of these trainings on the policymaking process in Nigeria, there is however little doubt that the knowledge and skill acquired by the participants, particularly the policymakers will improve their attitudes towards use of evidence in decision making process. Varkevisser and colleagues [45] observed in their study that capacity enhancement on health systems research (HSR) of policy-makers and other stakeholders in the health sector increased the national expertise for operational health research, and strengthen decision-making at all levels. In an earlier WHO expert consultation report [15], it was clearly noted that strengthening capacity for evidence-informed policymaking should involve both policymakers and researchers since capacity strengthening is needed for both researchers to generate better evidence and for policymakers and health-care professionals to better use available evidence. It is based on this premise that Dawad and Veenstra [13] argued that as researchers strive to develop the means to obtain timely information on health system impacts, policymakers need to be carried along to enable them become skilled at translating this information into appropriate action, to avoid forfeiting any progress made in developing and reforming the health system.

## Conclusion

In Nigeria, the grossly deficient capacity among policy-makers to use of evidence for policy-making remains a major challenge associated with evidence-to-policy link [36,39]. The promotion of evidence-informed policymaking cannot be adequately achieved without bridging the gap between researchers and policymakers. It is already well established that some differences exist between those who do research and those who may be in a position to use it. Some of these differences including include mutual mistrust and poor attitudes towards information among others have been found to persist largely due to the absence of opportunities to bring researchers, policy-makers together to consider issues around the research to policy and practice interface [30]. According to Green and Bennett [14] a major factor that can bridge the gaps in evidence-to-policy process is sufficient contact between researchers and policy-makers. Stressing on the need to promote the interaction between researchers and policy-makers, Choi and colleagues [30] noted that it is desirable for scientists and policy-makers to communicate their knowledge effectively or run the risks of barriers in language and understanding. They further noted that more incentives and opportunities to collaborate will help scientists and policy-makers appreciate their different goals, career paths, attitudes towards information, and perception of time. Long-term mechanisms that allow for periodic interactions between researchers and policymakers are therefore needed especially in LMICs. Studies show that establishing such long-term links between policymakers and researchers can result in greater involvement of policymakers in setting research priorities and increases the use of research [6].

### What is known about this topic

- There is currently a shift away from opinion-based policies to a more rigorous approach that uses high-quality research evidence to inform health policy-making;
- One of the most challenging issues with evidence-to-policy link is the capacity constraints of policymakers to access, synthesize, adapt and utilize available research evidence;
- Policy-makers need the capacity to be able to access and assess research findings and incorporate them in decision making.

### What this study adds

- Research on evidence-informed policymaking and knowledge transfer/exchange processes involving policymakers is new or still at infancy stage in Nigeria;
- Bringing researchers and policymakers together to participate in interventional training workshops can enhance their capacity for evidence informed policymaking;
- Capacity strengthening is needed for both researchers to generate better evidence and for policymakers and health-care professionals to better use available evidence.

## Competing interests

The authors declare no competing interests.

## References

[1] Loewenson R (2010). Connecting the streams: Using health systems research knowledge in low- and middle-income countries. Background paper for the global symposium on health systems research.

[2] Lavis JN, Davies HT, Oxman A, Denis JL, Golden-Biddle K, Ferlie E (2005). Towards systematic reviews that inform healthcare management and policymaking. J Health Serv Res Policy..

[3] Salchev P, Hristov N, Georgieva L Evidence Based Policy- Practical Approaches. The Bulgarian National Health Strategy 2007-2012..

[4] Campbell DM, Redman S, Jorm L, Cooke M, Zwi AB, Rychetnik L (2009). Increasing the Use of Evidence in Health Policy: Practice and Views of Policy Makers and Researchers. Austr New Zealand Health Pol..

[5] Dobrow MJ, Goel V, Upshur RE (2004). Evidence-Based Health Policy: Context and Utilisation. Soc Sci Med..

[6] Hanney SR, Gonzalez-Block MA, Buxton MJ, Kogan M (2003). The Utilization of Health Research in Policy-Making: Concepts, Examples and Methods of Assessment. Health Res Pol Systems..

[7] Innvaer S, Vist G, Trommald M, Oxman A (2002). Health Policy-Makers' Perceptions of their use of evidence: A Systematic Review. J Health Serv Res Pol..

[8] Travis P, Bennett S, Haines A, Pang T, Bhutta Z, Hyder AA, Pielemeier NR, Mills A, Evans T (2004). 2004 Overcoming Health-Systems Constraints to Achieve the Millennium Development Goals. Lancet..

[9] (2008). World Health Organization. Report on Meeting on Health Systems Strengthening and Primary Health Care. Report Series No RS/2008/GE/35(PHL)..

[10] World Health Organization (2004). World report on knowledge for better health: Strengthening health systems..

[11] Uneke CJ, Ezeoha AE, Ndukwe CD, Oyibo PG, Onwe F, Ogbonna A (2013). Assessment of organizational capacity for evidence-based health systems operations in Nigeria. Soc Work Public Health..

[12] González-Block MA, Mills A (2003). Assessing capacity for health policy and systems research in low and middle income countries. Health Res Policy Syst..

[13] Dawad S, Veenstra N (2007). Comparative health systems research in a context of HIV/AIDS: lessons from a multi-country study in South Africa, Tanzania and Zambia. Health Res Policy Syst..

[14] Green A, Bennett S (2007). Sound choices: enhancing capacity for evidence-informed health policy..

[15] World Health Organization (2008). Report: Consultation on strengthening health research capacity in the pacific..

[16] Lavis JN, Permanand G, Oxman AD, Lewin S, Fretheim A (2009). SUPPORT Tools for evidence-in-formed health policymaking (STP) 13: preparing and using policy briefs to support evidence-in-formed policymaking. Health Res Policy Syst..

[17] Lavis JN (2006). Moving forward on both systematic reviews and deliberative processes. Healthc Policy..

[18] Lomas J (2007). Decision support: a new approach to making the best healthcare management and policy choices. Healthc Q..

[19] Deans F, Ademokun A Investigating capacity to use evidence. International Network for the Availability of Scientific Publications (INASP)..

[20] Haines A, Kuruvilla S, Borchert M (2004). Bridging the implementation gap between knowledge and action for health. Bull World Health Organ..

[21] Etiaba E, Uguru N, Ebenso B, Russo G, Ezumah N, Uzochukwu B, Onwujekwe O (2015). Development of oral health policy in Nigeria: an analysis of the role of context, actors and policy process. BMC Oral Health..

[22] Hawkes S, K Aulakh B, Jadeja N, Jimenez M, Buse K, Anwar I, Barge S, Odubanjo MO, Shukla A, Ghaffar A, Whitworth J (2016). Strengthening capacity to apply health research evidence in policy making: experience from four countries. Health Policy Plan..

[23] Uneke CJ, Ndukwe CD, Ezeoha AA, Uro-Chukwu HC, Ezeonu CT (2015). Implementation of a health policy advisory committee as a knowledge translation platform: the Nigeria experience. Int J Health Policy Manag..

[24] Uneke CJ, Ezeoha AE, Ndukwe CD, Oyibo PG, Onwe F, Aulakh BK (2013). Research priority setting for health policy and health systems strengthening in Nigeria: the policymakers and stakeholders perspective and involvement. Pan Afr Med J..

[25] Moat KA, Lavis JN, Clancy SJ, El-Jardali F, Pantoja T (2014). Knowledge Translation Platform Evaluation study team. Evidence briefs and deliberative dialogues: perceptions and intentions to act on what was learnt. Bull World Health Organ..

[26] Uneke CJ, Ezeoha AE, Ndukwe CD, Oyibo PG, Onwe F (2013). Promotion of health sector reforms for health systems strengthening in Nigeria: perceptions of policy makers versus the general public on the Nigeria health systems performance. Soc Work Public Health..

[27] Uneke CJ, Ezeoha AE, Ndukwe CD, Oyibo PG, Onwe FD (2012). Enhancing leadership and governance competencies to strengthen health systems in Nigeria: assessment of organizational human resources development. Healthc Policy..

[28] Uneke CJ, Aulakh BK, Ezeoha AE, Ndukwe CD, Onwe F (2012). Bridging the divide between research and policy in Nigeria: the role of a health policy advisory committee. J Public Health Policy..

[29] Uneke CJ, Ezeoha AE, Ndukwe CD, Oyibo PG, Onwe F (2012). Promotion of evidence-informed health policymaking in Nigeria: bridging the gap between researchers and policymakers. Glob Public Health..

[30] Uneke CJ, Ezeoha AE, Ndukwe CD, Oyibo PG, Onwe F (2010). Development of health policy and systems research in Nigeria: lessons for developing countries' evidence-based health policy making process and practice. Healthc Policy..

[31] Okonofua F, Lambo E, Okeibunor J, Agholor K (2011). Advocacy for free maternal and child health care in Nigeria:Results and outcomes. Health Policy..

[32] Okonofua FE, Hammed A, Nzeribe E, Saidu B, Abass T, Adeboye G, Adegun T, Okolocha C (2009). Perceptions of policymakers in Nigeria toward unsafe abortion and maternal mortality. Int Perspect Sex Reprod Health..

[33] Garuba HA, Kohler JC, Huisman AM (2009). Transparency in Nigeria's public pharmaceutical sector: perceptions from policy makers. Global Health..

[34] Syed SB, Hyder AA, Bloom G, Sundaram S, Bhuiya A, Zhenzhong Z, Kanjilal B, Oladepo O, Pariyo G, Peters DH (2008). Future HealthSystems: Innovation for Equity: Exploring evidence-policy linkages in health research plans: a case study from six countries. Health Res Policy Syst..

[35] Sutcliffe S, Court J (2005). Evidence-Based Policymaking: What is it? How does it work? What relevance for developing countries?..

[36] Uneke CJ, Ezeoha AE, Ndukwe CD, Oyibo PG, Onwe F, Igbinedion EB, Chukwu PN (2011). Individual and organizational capacity for evidence use in policy making in Nigeria: an exploratory study of the perceptions of Nigeria health policy makers. Evidence Pol..

[37] Uneke CJ, Ezeoha AE, Ndukwe CD, Oyibo PG, Onwe F, Aulakh BK (2014). (2014). Enhancing policy makers? capacity for evidence-informed policy making through mentorship: A reflection on the Nigeria experience. Evidence Pol..

[38] Uneke CJ, Ezeoha AE, Uro-Chukwu H, Ezeonu CT, Ogbu O, Onwe F, Edoga C (2015). Promoting Evidence to Policy Link on the Control of Infectious Diseases of Poverty in Nigeria: Outcome of A Multi-Stakeholders Policy Dialogue. Health Prom Persp..

[39] Uneke CJ, Ezeoha AE, Uro-Chukwu H, Ezeonu CT, Ogbu O, Onwe F, Edoga C (2015). Enhancing the capacity of policy-makers to develop evidenceinformed policy brief on infectious diseases of poverty in Nigeria. Int J Health Pol Mgt..

[40] Healthcare for all (HIFA). CHILD2015 Summary: Are workshops effective? Available atPublished (2008).

[41] Poulos RG, Zwi AB, Lord SR (2007). Towards enhancing national capacity for evidence informed policy and practice in falls management: a role for a “Translation Task Group”. Aust New Zealand Health Policy..

[42] Choi BC, Gupta A, Ward B (2009). Good thinking: six ways to bridge the gap between scientists and policy makers. J Epidemiol Comm Health..

[43] Choi BC, McQueen DV, Rootman I (2003). Bridging the gap between scientists and decision makers. J Epidemiol Comm Health..

[44] Choi BC, Pang T, Lin V, Puska P, Sherman G, Goddard M, Ackland MJ, Sainsbury P, Stachenko S, Morrison H, Clottey C (2005). Can scientists and policy makers work together?. J Epidemiol Comm Health..

[45] Varkevisser CM, Mwaluko GM, Le Grand A (2001). Research in action: the training approach of the Joint Health Systems Research Project for the Southern African Region. Health Policy Plan..

